# Factors associated with discriminatory attitudes towards people living with HIV among adult population in Ethiopia: analysis on Ethiopian demographic and health survey

**DOI:** 10.1080/17290376.2020.1857300

**Published:** 2020-12-23

**Authors:** Gedefaw Alen Diress, Mohammed Ahmed, Melese Linger

**Affiliations:** Department of Public Health, College of Health Science, Woldia University, Woldia, Ethiopia

**Keywords:** Stigma, discrimination, HIV, Ethiopia

## Abstract

Extensive discriminatory attitudes in a population can affect people’s willingness to be tested for Human Immunodeficiency Virus (HIV), their initiation of antiretroviral therapy, social support as well as the quality of life of people infected with HIV. This study aimed to assess factors associated with discriminatory attitudes towards people living with HIV/AIDS (PLWHA). Secondary data analysis was conducted using data from the 2016 Ethiopia Demographic Health Survey. A total of 26,623 adult populations were included. Multivariable logistic regression analysis was conducted to identify factors associated with discriminatory attitudes. The proportion of participants having discriminatory attitudes towards PLWHA was 93.8% among men and 64.5% among women. This study revealed that rural residence, no formal education, lack of media access, not previously tested for HIV and lack of comprehensive HIV knowledge increase the odds of having discriminatory attitudes. In conclusion, there is a high-level discriminatory attitude towards PLWHA. Improvement in HIV-related knowledge and dealing with wrong perceptions and myths are extremely vital to reduce discriminatory attitudes towards HIV-infected people. Information, education and communication programmes need to intensify its educational campaigns to dispel these misconceptions.

## Introduction

In Ethiopia, the national HIV prevalence in 2017 was 1.16% and an estimated 722,248 people are currently living with HIV (‘National guidelines for comprehensive HIV prevention care and treatment, Ethiopia, MOH,’ 2017). Stigma and discriminatory attitudes are key elements that should be taken into account to inform effective HIV programming. HIV-related stigma refers to negative beliefs, feelings, attitudes and perceptions towards people living with HIV (PLHIV), while discrimination is a differential action or behaviour towards the stigmatised person based on those attitudes and perceptions (USAID, 2006). In Sub-Saharan countries, HIV is transmitted primarily through sexual intercourse. Therefore, in these countries, including Ethiopia, HIV is widely viewed as a consequence of sexual immoral behaviours; thus, PLHIV are severely stigmatised regardless of how they actually became infected (Nachega et al., 2012).

Previous evidence in different parts of Africa revealed that the magnitude of HIV-related stigma and discriminatory attitudes were significantly high. In Botswana, 60% of individuals stated that they would not buy vegetables from a shopkeeper with HIV/AIDS (Letamo, 2003). In Nigeria, around 40% of the population agreed that a female teacher infected with HIV should not be allowed to continue teaching and about 50% would not buy vegetables from vendors with HIV infection (Dahlui et al., 2015). Similarly, a study done in Ghana showed that 29% of people agreed that people with HIV should be isolated in certain villages or towns (Ulasi et al., 2009). In Ethiopia, based on the 2011 EDHS, 72.1% of the rural and 34.2% of the urban women had discriminatory and stigmatising attitudes towards people living with HIV (Gurmu & Etana, 2015).

Extensive stigma and discrimination in a population can affect people’s willingness to be tested for HIV, (Central Statistical Agency (CSA) [Ethiopia] & ICF, 2016; Chimoyi et al., 2015) their initiation of and adherence to antiretroviral therapy (ART), social support (Asamoah, Asamoah, & Agardh, 2017; Central Statistical Agency (CSA) [Ethiopia] & ICF, 2016; Katz et al., 2013; Ulasi et al., 2009) as well as the quality of life of people infected with HIV (Nattabi, Li, Thompson, Orach, & Earnest, 2012). In addition, it prevents individuals from disclosing their status even to family members and sexual partners and/ or accessing medical care and treatment, weakening their ability to protect themselves from getting or transmitting HIV and to stay healthy (Arrey, Bilsen, Lacor, & Deschepper, 2017; Simbayi et al., 2006). It might also affect the desire to have children among PLHIV, which results in emotional stress (Nattabi et al., 2012).

Even discrimination is experienced in a health-care setting by health-care providers who are responsible for stigma and discrimination reduction, which might exacerbate negative health outcomes (Batey et al., 2016). Discriminatory attitudes in health-care providers and the general population can affect people’s desire to know their status and enrol in HIV chronic care. Thus, low levels of discriminatory attitudes in a community are substantial indicators of the achievement of programmes that targeting HIV/AIDS prevention and control like improving HIV testing (Young et al., 2010).

Studies have shown that different factors such as educational status, age, employment (Ulasi et al., 2009), previous history of HIV testing, knowledge of ARTs and communication regarding HIV/AIDS (Genberg et al., 2009) can affect stigma and discrimination. Poor comprehensive knowledge to HIV and its ways of transmission are a critical sources of stigma and discrimination against people infected with HIV in Sub-Saharan Africa (Tarkang, Pencille, & Komesuor, 2018). A qualitative study conducted in Uganda showed that gender, family relationships and socio-economic factors appeared as important drivers of stigma and stigmatisation (Mburu et al., 2013). A systematic review done in Africa suggested that to have maximum effectiveness on improving ART adherence, there must be interventions to reduce stigma and discrimination after identifying the barriers (Katz et al., 2013).

Health-care providers play an important role in reducing stigma and discrimination with community-level interventions, care and support. HIV chronic care and treatment requires broad support for people living with HIV from their health-care teams to stay in care, adhere to treatment and cope with stigma and discrimination. However, in Ethiopia, access to chronic care and ARTs were limited which resulted in stigma and discrimination to be a substantial problem. In response, the most recent Ethiopian national guidelines for comprehensive HIV prevention care and treatment has called for research to identify factors affecting discriminatory attitudes and stigma, to inform future policies, culturally appropriate interventions, and stigma and discrimination reduction initiatives (National consolidated guidelines for comprehensive HIV prevention, care and treatment, 2018; Wube, Horne, & Stuer, 2010).

The factors associated with discriminatory attitudes towards people living with HIV/AIDS (PLWHA) in the general population of Ethiopia have not previously been explored at any depth at the national level with a large sample size. This paper is a first step towards filling this gap by providing information on factors that affect discriminatory attitudes. Therefore, the objective of this study was to identify the determinants of discriminatory attitudes towards PLWHA, which can give important inputs for the reduction of stigma and discrimination in Ethiopia.

## Methods

This study used secondary data from the 2016 EDHS. A detailed description of the study design and methodology of EDHS is found somewhere else (Central Statistical Agency (CSA) [Ethiopia] & ICF, 2016). All women aged 15–49 and all men aged 15–59 who were either permanent residents of the selected households or visitors who stayed in the household a night before the survey were eligible to be interviewed. Data were obtained from the DHS programme website. A total of 12,688 men aged 15–59 years and 15,683 women aged 15–49 participated in the survey. However, in this paper, only the respondents had ever heard of AIDS and responded to two discriminatory attitude questions (Would you buy vegetables from a vendor with HIV and children with HIV should be allowed to attend school with children without HIV) were included. Therefore, we restricted our analytical sample to 12,254 men aged 15–49 years and14, 369 women aged 15–49 years.

### Study variables and measurement

In the 2016 EDHS, there were questions on stigma and discrimination towards PLWHA. In the survey, discriminatory attitude towards PLWHA was measured using the following two questions: (1) Would you buy vegetables from a vendor with HIV and (2) Children with HIV should be allowed to attend school with children without HIV. One binary outcome variable (discriminatory attitude (yes/no)) was computed from the above two questions. Participants who responded ‘no’ or ‘I don’t know’ for at least one question were categorised as ‘having discriminatory attitude’, whereas participants who responded ‘yes’ for both questions were categorised as ‘having non-discriminatory attitude’.

The independent variables include age, residence, educational status, wealth index, access to media, internet use, substance use, comprehensive knowledge to HIV and number of lifetime sexual partners.

Access to media (media exposure) was labelled based on response to how often respondents read a newspaper, listened to the radio or watched television. Those who responded at least once a week to any of these sources were considered to have access to media. Substance use was defined based on response to whether or not participants chew *chat/Catha edulis* (leaves that are the source of habituating stimulant when chewed), drink alcohol or smoke cigarette. In this analysis, participants were classified as having comprehensive HIV knowledge if they correctly responded all of the following 5 questions: (1) Consistent use of condoms during sexual intercourse can reduce the chance of getting HIV, (2) Having just one uninfected faithful partner can reduce the chance of getting HIV, (3) Healthy-looking person can have HIV, (4) HIV can be transmitted by mosquito bites and (5) A person can become infected by sharing food with a person who has HIV.

### Statistical analysis

Statistical Package for the Social Sciences (SPSS) version 20 with complex samples procedure was used for the analysis. Multivariable logistic regression analysis was conducted to control confounders and identify the factors associated with discriminatory attitudes. All independent variables were entered in the multivariable regression model irrespective of the *p*-values at bivariate analysis. Adjusted odds ratios (AORs) with 95% CI were used to declare statistically significant association. All statistical procedures incorporated a complex sampling design applied in the 2016 EDHS.

## Results

### Socio-demographic and health-related characteristics of participants

In this analysis, a total of 26,623 adult populations (12,254 men and 14,369 women) were included. The majority of participants (38.5%) were between the age of 15 and 24 years and 66.1% of the participants were rural residents. The majority of the study participants (49.2%) were married and nearly one-fourth of participants did not have any formal education. In this study, 54% of them had access to media and only 29.0% of participants had comprehensive knowledge to HIV. More than half (46.6%) of the respondents were ever tested for HIV in their life (Table 1).

**Table 1.** Background information of participants (*N* =  26,623).

	Frequency (%)		
Variables	Men (*n* = 12,254)	Women (*n* = 14,369)	Total (*n* = 26,623)
Age			
15–24	4292 (34.6)	5962 (39.4)	10,254 (38.5)
25–34	3521 (28.6)	4652 (33.9)	8173 (30.7)
35–49	3360 (28.2)	3791 (26.7)	7151 (26.8)
50–59	1081 (8.6)	–	1081 (4.0)
Residence			
Urban	3806 (19.9)	5232 (23.5)	9038 (33.9)
Rural	8448 (80.1)	9137 (76.5)	17,585 (66.1)
Marital status			
Unmarried	4882 (37.9)	4457 (28.9)	9339 (35.0)
Married	7010 (57.2)	8682 (63.5)	15,692 (49.2)
Widowed	55 (0.45)	401 (2.7)	456 (1.64)
Divorce	307 (2.51)	829 (4.9)	1136 (4.26)
Educational status			
No education	3266 (29.8)	5969 (45.4)	1348 (23.2)
Primary	5177 (46.5)	4994 (36.2)	2260 (37.1)
Secondary	2212 (14.8)	2214 (12.4)	1618 (25.0)
Higher	1599 (8.9)	1192 (6.0)	1027 (14.7)
Employment			
Unemployed	5232 (42.7)	7149 (49.1)	12,381 (46.4)
Employed	7022 (57.3)	7220 (50.9)	14,242 (53.6)
Wealth index			
Poorest	2650 (15.5)	3097 (15.3)	5747 (21.6)
Poorer	1757 (18.2)	1844 (17.3)	3601 (13.5)
Middle	1691 (19.2)	1861 (18.9)	3552 (13.3)
Richer	1889 (21.7)	1950 (20.3)	3839 (14.4)
Richest	4267 (25.4)	5617 (28.2)	9884 (37.1)
Access to media			
Yes	4951 (40.4)	7386 (45.9)	12,337 (46.3)
No	7303 (59.6)	6983 (54.1)	14,286 (53.7)
Internet use			
Yes	1596 (13.2)	1347 (5.3)	2943 (11.0)
No	10,658 (86.8)	13,022 (94.7)	23,680 (89.0)
Comprehensive knowledge to HIV			
Yes	3970 (32.4)	3731 (25.2)	7743 (29.0)
No	8284 (67.6)	10,638 (74.8)	18,922 (71.0)
Ever tested for HIV			
Yes	4791 (39.1)	7602 (47.5)	12,393 (46.6)
No	7463 (60.9)	6767 (52.5)	14,230 (53.4)

Note: Frequency (*n*) =  unweighted count percentage (%) = weighted percent.

### Magnitude of discriminatory attitudes towards HIV-infected people

In this study, the overall magnitude of discriminatory attitudes towards HIV-infected people among the general population was 74.7%. However, there was a significant difference between men and women (*p* < .05). The proportion of participants having discriminatory attitudes towards PLWHA was 93.8% among men and 64.5% among women. About 43.5% of women and 49.1% of men would not buy vegetables from vendors with HIV infection. Similarly, 62.4% of men and 49.2% of women agreed that children with HIV should not be allowed to attend school with children without HIV (Table 2).

**Table 2.** Discriminatory attitude towards HIV-infected people by gender.

Variables		Women	Men	^a^*p*-value
Children with HIV should be allowed to attend school with children without HIV	Yes	7069 (49.2)	7647 (62.4)	.02
	No	7300 (50.8)	4607 (37.6)	
Would you buy vegetables from a vendor with HIV	Yes	6251 (43.5)	6017 (49.1)	.14
	No	8118 (56.5)	6237 (50.9)	
Over all discriminatory attitude towards HIV-infected people	Yes	13,478 (93.8)	7904 (64.5)	<.001
	No	891 (6.2)	4350 (35.5)	

^a^Significance level of chi-square (*χ*^2^).

### Factors associated with discriminatory attitudes

In this analysis, multivariable logistic regression was employed. All independent variables were entered in the multivariable regression model irrespective of the *p*-values at bivariate analysis. The AORs and 95% CIs are presented in Table 3.

**Table 3.** Bi-variable and multivariable analysis on factors associated with discriminatory attitude among adult population Ethiopia.

Variables	COR (95% CI)	AOR (95% CI)
Age		
15–24	0.54 (0.468–0.626)	1.04 (0.870–1.246)
25–34	0.78 (0.682–0.894)	1.01 (0.861–1.174)
35–59	1	1
Residence		
Urban	1	
Rural	7.14 (5.52–9.34)	1.89 (1.24–2.67)**
Marital status		
Single	Ref	Ref.
Married	2.67 (2.325–3.062)	1.51 (0.832–1.767)
Divorce	1.42 (1.124–1.790)	0.95 (0.730–1.225)
Widowed	1.40 (0.968–2.024)	0.73 (0.479–1.098)
Educational status		
No education	Ref.	5.88 (4.10–8.33)*
Primary	0.39 (0.334–0.446)	1.72 (0.60–2.02)
Secondary	0.09 (0.068–0.111)	4.0 (3.16–5.24)*
Higher	0.03 (0.024–0.046)	1
Employment		
Unemployed	1.34 (1.158–1.551)	0.98 (0.850–1.141)
Employed	1	1
Wealth index		
Poorest	9.86 (7.509–12.946)	2.02 (0.477–2.751)
Poorer	7.80 (6.184–10.342)	1.89 (0.414–2.530)
Middle	6.03 (4.803–7.568)	1.57 (0.206–2.049)
Richer	3.84 (3.023–4.884)	1.25 (0.944–1.662)
Richest	Ref.	Ref
Access to media		
No	3.70 (3.09–4.39)	1.56 (1.23–2.00)**
Yes	1	1
Internet use		
No	1	1
Yes	0.07 (0.052–0.091)	0.47 (0.341–1.634)
Ever tested for HIV		
No	2.63 (2.29–3.02)	1.72 (1.48–2.01)*
Yes	1	1
Comprehensive HIV knowledge		
No	1.54 (1.13–4.67)*	1.96 (1.70–2.26)**
Yes	1	1

*p-value <.05.

**p-value < .001.

The results of this study showed that residence, educational status, previous history of HIV test, access to media and comprehensive knowledge to HIV were significantly associated with discriminatory attitude towards PLWHA after controlling for other predictors in the model. It was found that participants with rural residence had 89% higher odds of having discriminatory attitudes when compared with urban (AOR =  1.89 (95% CI:1.24–2.67).

With regard to educational status, the odds of having discriminatory attitude increased significantly with no formal education (AOR =  5.88 (95%CI: 4.10–8.33)] and secondary level of education (AOR = 4.0 (95%CI: 3.16–5.24) as compared to those with higher educational level. Similarly, participants who had no access to media were more likely to have discriminatory attitude towards HIV-infected people (AOR =  1.56 (95% CI: 1.23–2.00)). Individuals who had not ever been tested for HIV (AOR =  1.72 (95% CI: 1.48–2.00)) and those had no comprehensive HIV knowledge (AOR =  1.96 (95% CI: 1.70–2.26)) were more likely to show discriminatory attitude towards PLWHA when compared with their counterpart (Table 3).

### Factors associated with acceptance towards vendor infected with HIV

People those living in urban locations, those with higher, secondary or primary level of education, those who are married, those using internet and those who have good comprehensive HIV knowledge tend to agree more that they would buy vegetables from a vendor with HIV. Individuals who were in urban residence were 54% less likely than those living in rural to indicate that they would not buy vegetables from a vendor with HIV [AOR =  0.46 (95% CI: 0.313–0.690)]. Married participants were 1.4 times more likely than those single to state that they would not buy vegetables from a vendor with HIV (AOR =  1.36 (95% CI:1.167–1.579)). Individuals with primary education were 37% less likely than those with no education to indicate that they would not buy vegetables from a vendor with HIV (AOR =  0.63 (95% CI: 0.542–0.723)). Again, person with higher education were 85% less likely than those with no education to indicate that they would not buy vegetables from a vendor with HIV (AOR =  0.15 (95% CI: 0.089–0.242)). Participants who had comprehensive HIV knowledge were 46% less likely than those who had no comprehensive HIV knowledge to state that they would not buy vegetables from a vendor with HIV (AOR =  0.54 (95% CI:0.470–0.617)) (Table 4).

**Table 4.** Multivariate analysis for each separate outcome variables (Would not buy vegetables from vendor with HIV or Children with HIV should not be allowed to attend school with children without HIV)

Discriminatory attitude		
Variables	Would not buy vegetables from vendor with HIV	Children with HIV should not be allowed to attend school with children without HIV
	AOR (95%CI)	AOR (95% CI)
Age		
15–24	1.11 (0.934–1.311)	0.867 (0.729–1.031)
25–34	1.07 (0.927–1.240)	1.04 (0.883–1.165)
35–59	1	1
Residence		
Urban	0.46 (0.313–0.690)**	0.65 (0.466–0.863)**
Rural	1	1
Marital status		
Single	Ref	Ref.
Married	1.36 (1.167–1.579)*	1.51 (0.832–1.767)
Divorce	0.83 (0.561–1.214)	0.45 (0.130–1.015)
Widowed	0.91 (0.714–1.171)	0.63 (0.579–1.098)
Educational status		
No education	Ref.	Ref.
Primary	0.63 (0.542–0.723)*	0.63 (0.540–1.025)
Secondary	0.26 (0.199–0.326)*	0.86 (0.198–1.337)
Higher	0.15 (0.089–0.242)*	0.23 (0.159–0.325)*
Employment status		
Unemployed	0.88 (0.767–1.011)	1.24 (1.050–1.541)
Employed	1	1
Wealth index		
Poorest	1.86 (0.509–12.946)	1.35 (0.837–2.611)
Poorer	1.28 (0.841–2.342)	1.83 (0.614–2.530)
Middle	1.13 (0.803–1.568)	1.57 (0.206–2.049)
Richer	1.06 (0.539–1.884)	1.25 (0.944–1.662)
Richest	Ref.	Ref
Access to media		
No	Ref.	Ref.
Yes	0.85 (0.721–1.012)	0.64 (0.501–0.814)**
Internet use		
No	1	1
Yes	0.44 (0.308–0.619)	0.36 (0.241–1.047)
Ever tested for HIV		
No	Ref	Ref.
Yes	0.67 (0.577–1.170)	0.78 (0.599–1.216)
Comprehensive HIV knowledge		
No	1	1
Yes	0.54 (0.470–0.617)**	0.47 (0.400–0.556)*

*p-value < .05.

**p-value < .001.

### Factors associated with acceptance towards children infected with HIV

Participants living in urban, those with higher level of education, those who have media access and those who have good comprehensive knowledge to HIV tend to agree more that children with HIV should be allowed to attend school with children without HIV tested for HIV. Individuals living in urban residence were 35% less likely than those living in rural to indicate that children with HIV should not be allowed to attend school with children without HIV (AOR =  0.65 (95% CI:0.466–0.863)). Participants with higher education were 77% less likely than those with no education to indicate that children with HIV should not be allowed to attend school with children without HIV (AOR =  0.23 (95% CI:0.159–0.325)). Participants who had media access were 36% less likely than those who had no access to media to indicate that children with HIV should not be allowed to attend school with children without HIV (AOR =  0.64 (95% CI:0.501–0.814)). Individuals who had comprehensive HIV knowledge were 53% less likely than those who had no comprehensive HIV knowledge to indicate that children with HIV should not be allowed to attend school with children without HIV (AOR =  0.47 (95% CI:0.400–0.556)) (Table 4.)

## Discussion

The findings in this study suggest that there is a high level of discriminatory attitude towards HIV-infected people. This is consistent with previous studies (Dahlui et al., 2015; Young et al., 2010). Consistent with previous studies, stigma appears to be quite high in this sample, with about 43.5% of women and 49.1% of men not willing to buy vegetables from a vendor with HIV as was found in Nigeria (Dahlui et al., 2015) but a little bit lower than studies done in Botswana and South Africa (Letamo, 2003; Mall, Middelkoop, Mark, Wood, & Bekker, 2013). This high level of discriminatory attitude might have substantial adverse effects on the day-to-day lives of HIV-infected people. It might result in emotional stress, inconsistent health-care-seeking behaviour and non-disclosure of HIV status (Arrey et al., 2017; Simbayi et al., 2006). Besides, it may lead to inadvertent transmission of the virus, inadequate self-care, difficulties with safer sex negotiation and condom use and leads some to avoid HIV testing (Chimoyi et al., 2015). It can also affect their initiation of and adherence to ART and social support (Central Statistical Agency (CSA) [Ethiopia] & ICF, 2016; Katz et al., 2013). Subsequently, high levels of discriminatory attitude create the circumstances for spreading HIV and undermine the ability of individuals and communities to protect them which is one difficulty in achieving the three 90s in Ethiopia (by 2020, 90% of all people living with HIV will know their HIV status, 90% of all people with diagnosed HIV infection will receive sustained antiretroviral therapy and 90% of all people receiving antiretroviral therapy will have viral suppression).

In this study residence, educational status, media access, ever tested for HIV and comprehensive HIV knowledge were significantly associated with stigma and discrimination towards HIV-infected people. Consistent with the previous literature, we found that individuals who lived in rural residence were more likely to show stigma and discriminatory attitudes when compared with their counterparts (Dahlui et al., 2015; Letamo, 2003). Individuals who lived in urban have access of various mass media (television, radio, newspaper, etc.) which is a powerful way of sending public health and health promotion messages to the population and particularly for sending stigma- and discrimination-related messages to the community.

In this study, people with a low level of educational status were associated with HIV-related stigma and discriminatory attitudes which supported with past studies (Dahlui et al., 2015; Haffejee, Maughan-Brown, Buthelezi, & Kharsany, 2018; Letamo, 2003). Non-educated individuals might not have an understanding of the disease (including misconceptions about modes of transmission) and consequence of discriminatory attitudes which might result in increased HIV-related stigma and discriminatory attitudes towards HIV-infected people.

Participants who had no media access were more likely to express a discriminatory attitude towards HIV-infected people. This is consistent with previous studies that reported media as one of the important sources of HIV/AIDS information in terms of prevention and decreasing stigmatising behaviour (Asamoah et al., 2017; LaCroix, Snyder, Huedo-Medina, & Johnson, 2014; Romer et al., 2009). Media (television, radio and newspaper), especially television, is an influential way of transmitting public health and health promotion sensitive messages as well as being able to effectively change health behaviours in individuals with low literacy levels who otherwise might find it difficult to process such messages (LaCroix et al., 2014; Singhal & Rogers, 1999).

In the current study, individuals who had not been tested for HIV were more likely to express discriminatory attitudes towards HIV-infected people. Individuals might gain HIV-related information during counselling at the time HIV testing which may reduce negative attitudes about HIV-infected people which is supported with previous studies (Genberg et al., 2009; Hutchinson & Mahlalela, 2006; Pulerwitz, Michaelis, Lippman, Chinaglia, & Díaz, 2008). Similar to previous studies (Asamoah et al., 2017; Mall et al., 2013), discriminatory attitudes were high among participants who have no comprehensive HIV knowledge. This shows that the absence of realistic information about the means of transmission of HIV and the corresponding myths associated with AIDS transmission contribute to the stigmatising behaviours and discriminatory attitudes towards HIV-infected people.

Accordingly, the major contribution of the current study is that it provides empirical data on the magnitude of discriminatory attitudes towards PLHIV at the national level. This study also identifies important factors associated with discriminatory attitudes in the general population. This will allow policymakers, non-governmental organisations and other stakeholders to design stigma and discrimination reduction initiatives.

This study has several strengths, which include relatively large sample size, nationally representative nature, availability of detailed data on confounders and standardised, high-quality data collection. However, there are limitations to consider. Firstly, as cross-sectional data were used, we cannot assign causality. Data were collected based on self-report which might result in social desirability. Finally, even all known confounding factors are controlled for using multivariable analysis, the estimated associations between the outcome and the exposure can be affected by unmeasured confounding (residual confounding). Therefore, an observed significant association provides limited evidence for a causal relationship.

## Conclusion

In conclusion, residence, educational status, media access, ever tested for HIV and comprehensive HIV knowledge were significantly associated with a discriminatory attitude towards HIV-infected people. Improvement in a comprehensive knowledge of HIV like routes of transmission and prevention methods as well as dealing with wrong perceptions and myths are extremely vital to reduce discriminatory attitudes towards PLWHA. Therefore, the information, education and communication programme (increase media utilisation focusing on stigma and discrimination) needs to intensify its educational campaigns to dispel these misconceptions. In addition, expanding access to HIV counselling and testing services should be done and educational programs need to be designed to change discriminatory attitudes.

## Data Availability

The data can be available from the corresponding author and DHS database.

## Abbreviations

**Abbreviations**: AOR: adjusted odds ratio; COR: crude odds ratio; EDHS: Ethiopia demographic health science; HIV: human immune-deficiency virus; PLWHA: people living with HIV/AIDS; SPSS: Statistical Package for Social Science
